# A Unique Case of Acute Coronary Syndrome in a Patient With COVID-19 Infection

**DOI:** 10.7759/cureus.15650

**Published:** 2021-06-14

**Authors:** Juwairiya Shuroog, Joseph Raffetto, Shahabuddin Soherwardi, Maleeha Hassan, Simona Eng, Fahad Nayim

**Affiliations:** 1 Internal Medicine, TidalHealth Peninsula, Salisbury, USA; 2 Cardiology, TidalHealth Peninsula, Salisbury, USA; 3 Internal Medicine, West Virginia University School of Medicine, Morgantown, USA

**Keywords:** covid 19, microthrombi, nste-acs, hyper coagulopathy, covid and cardiovascular complications

## Abstract

The coronavirus pandemic has caused significant mortality and morbidity in just over a year of its course since the first case was identified in Wuhan, China in December 2019. The varied presenting symptoms of this enveloped positive-sense single-stranded RNA virus infection and the unknown surrounding the pathophysiology of the disease process have been extensively reported in the literature. In this case report, we present a coronavirus disease 2019 (COVID-19) positive patient who presented with chest pain, diagnosed with acute coronary syndrome. Interestingly, the patient was noted to have non-ST elevation myocardial infarction with cardiac catheterization showing coronary microthrombi rather than typical acute coronary thrombotic occlusive disease.

## Introduction

The global pandemic of coronavirus disease caused by COVID-19 virus has resulted in significant mortality and morbidity worldwide with a substantial majority of hospitalized patients developing acute hypoxic respiratory failure and a proportion of which also developed acute respiratory distress syndrome. It is estimated that up to a quarter of the hospitalized patient also developed major cardiovascular complications from severe COVID-19 infection [1,2]. Presentation varies from cardiomyopathy, obstructive coronary artery disease, ventricular arrhythmias, and myocarditis [2]. The prognosis of such patients is poor as compared to those COVID-19 patients without cardiovascular complications [3].

## Case presentation

**Figure 1:**
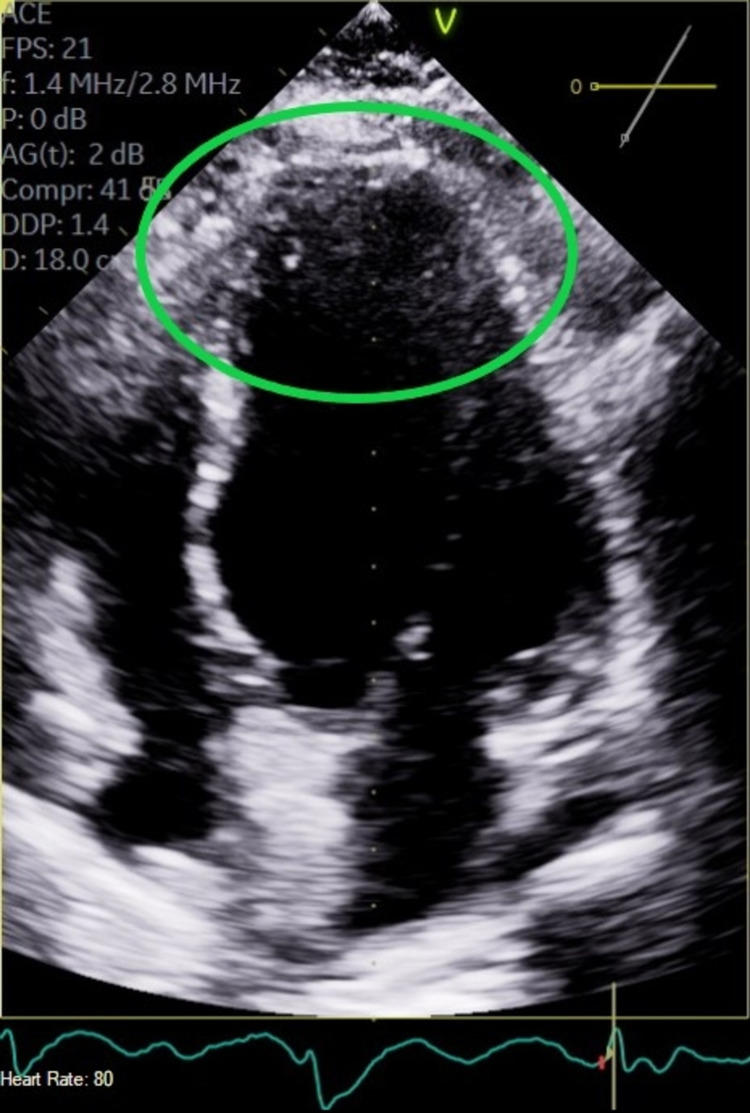
End diastolic phase (green oval).

**Figure 2:**
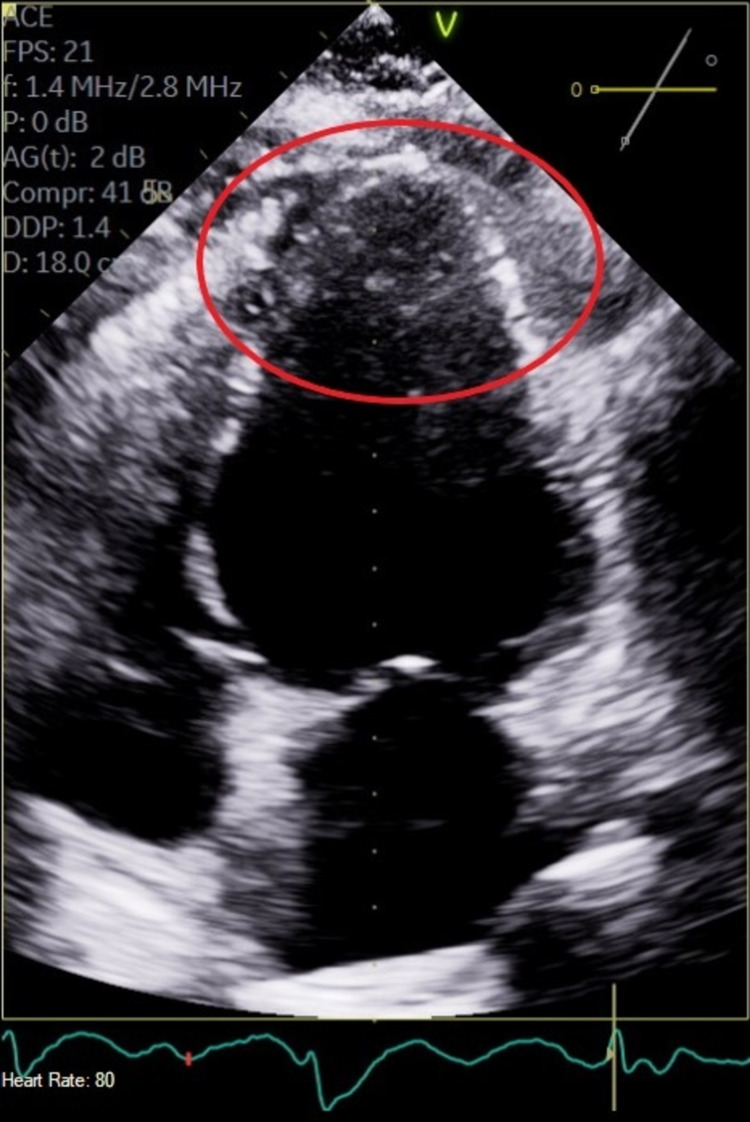
End systolic phase showing anterior septal hypokinesia (red oval).

**Figure 3:**
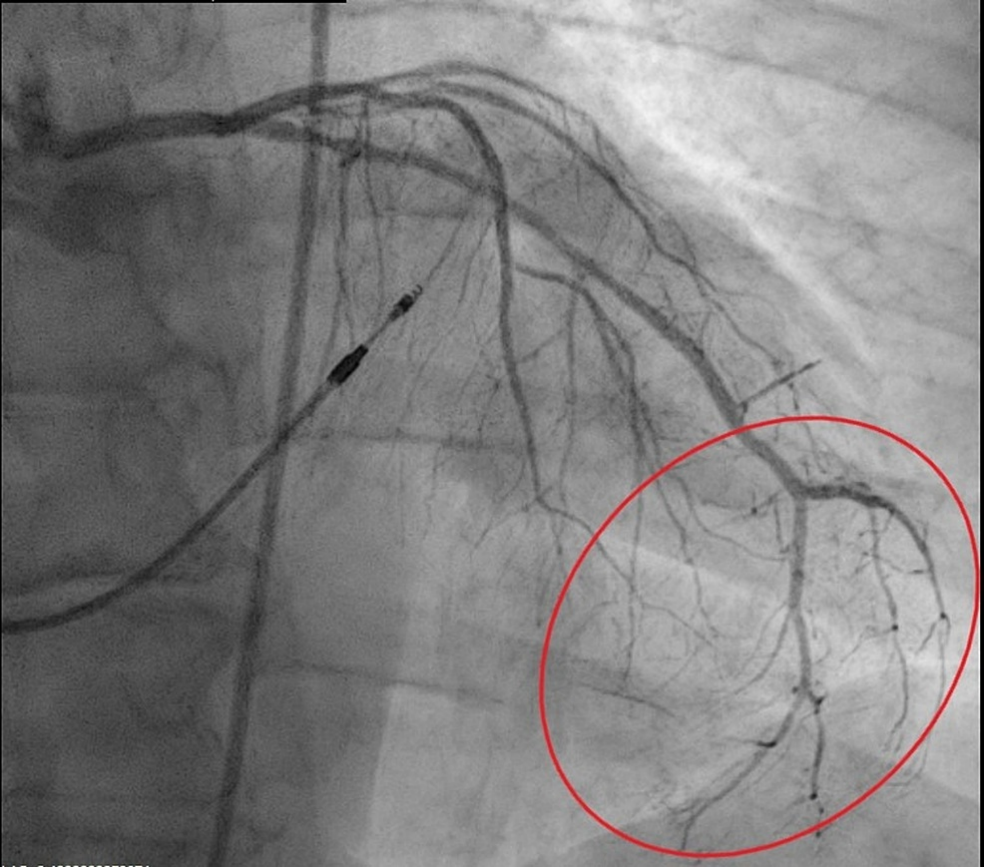
Cardiac catheterization image showing multiple luminal irregularities in left anterior descending artery tertiary with flow cut off at distal branches (red oval).

**Figure 4:**
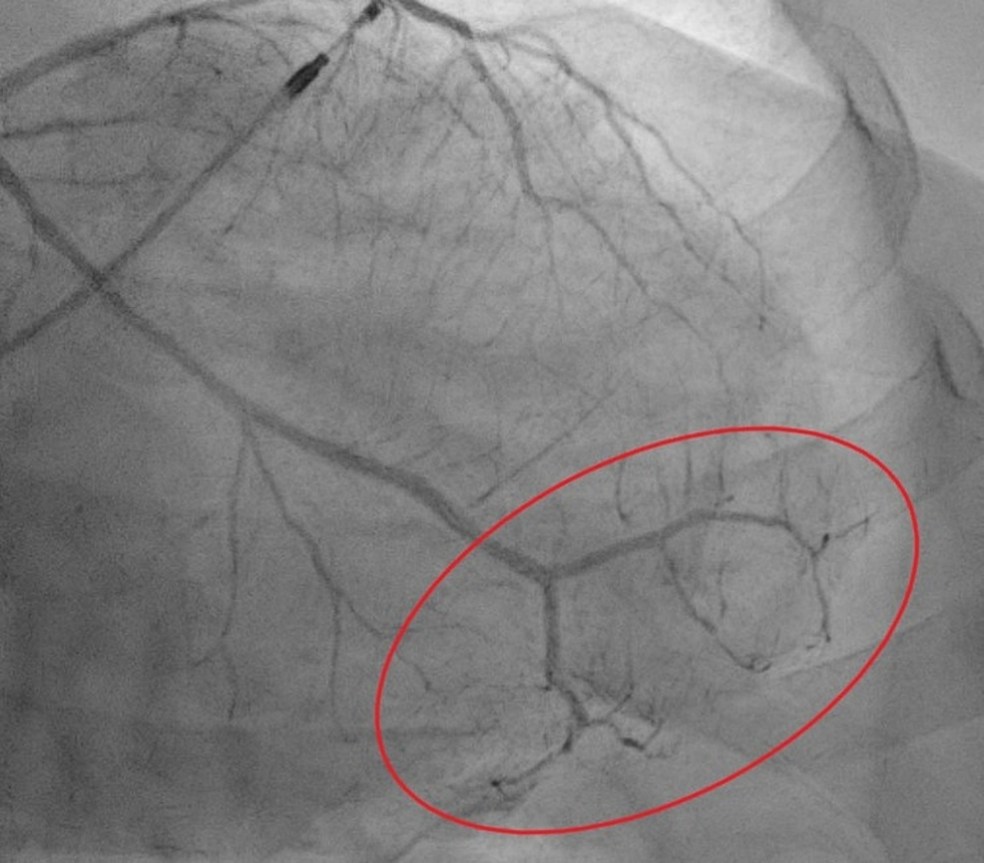
Cardiac catheterization image showing multiple luminal irregularities in left anterior descending artery tertiary with flow cut off at distal branches (red oval).

This is a 72-year-old female with a past medical history of complete heart block (status post permanent pacemaker), chronic tobacco smoker, history of coronary artery disease, who presented with intermittent chest pain for three-day duration which worsened with exertion and relieved with rest. Initial workup showed ST depression in lead I/aVL/V5/V6. The patient also had elevated troponin of 5.2 ng/mL which up trended to a peak of 15.44 ng/mL within 24 hours. The lipid panel was abnormal with an elevated triglyceride of 161 mg/dL, low-density lipoprotein (LDL) elevated at 148 mg/dL. D-dimer was within normal limits. The patient was started on acute coronary syndrome protocol for non-ST elevation MI. Transthoracic echocardiogram showed an ejection fraction (EF) of 45% calculated with Simpson's rule, there were regional wall motion abnormalities noted with moderate anterior septal hypokinesia (Figures 1, 2). Chest pain resolved following nitroglycerin as well as initiation of therapeutic anticoagulation with enoxaparin. Interestingly preprocedural screening for COVID-19 came back positive, cardiac catheterization showed angiographically normal left coronary arterial system with evidence of minimal atherosclerotic plaquing of the mid aspect of left anterior descending (LAD) as well as normal right coronary artery (RCA) with minimal atherosclerotic plaquing of the proximal segment of the vessel. Of note, angiographic evaluation of the microvasculature appeared quite prominent with evidence of luminal irregularities and evidence of small vessel disease with flow cut off at distal branches. Best interpreted as microthrombi as theses were noted in multiple views and were consistent (Figures 3, 4). Prior cardiac catheterization about two years ago had no evidence of coronary artery disease and did not have any luminal irregularities. The patient was medically managed with oral anticoagulation therapy with patient remaining symptom-free at one-month follow-up

## Discussion

The Joint Task Force of the European Society of Cardiology, American College of Cardiology Foundation, the American Heart Association, and the World Heart Federation (ESC/ACCF/AHA/WHF) defined acute myocardial infarction (MI) (2018) as the presence of acute myocardial injury detected by abnormal cardiac biomarkers in the setting of acute myocardial ischemia [4], defining five clinical classifications of MI. Primarily type I MI is caused by acute atherosclerotic coronary artery disease and usually precipitated by atherosclerotic plaque disruption. However, type II MI is a classification defined by a mismatch between oxygen supply and demand with potential mechanisms being microvascular dysfunction, emboli, vasospasm, coronary dissection as well as conditions resulting in increased myocardial oxygen demand. The case presented in this article represents a case of type II MI. In the majority of these cases, patients are managed medically with the treatment of underlying etiology, which in our case was anticoagulation for coronary microthrombi.

Prior experience with acute viral myocarditis has shown that cardiotropic viral entry into the myocyte may result in direct cellular injury to the myocyte. Subsequently, a patient-mediated immune response can elicit profound inflammatory activation and cytokine release resulting in focal or diffuse myocardial necrosis. The pathogenesis of cardiac involvement in COVID-19 infection may be associated either with the dissemination of the virus to the lymphatic system from the respiratory tract or through the bloodstream. This is similarly noted as significant inflammation in the alveoli of patients with acute respiratory distress syndrome (ARDS) associated with COVID-19 pneumonia and exaggerated inflammatory response may also be contributing to myocardial injury justifying the use of corticosteroids to attenuate inflammation which is currently widely practiced [5]. This is also supported by dexamethasone use in the recovery trial.

The underlying mechanisms for type 2 MI are further elucidated by postmortem evaluation of individuals afflicted with COVID-19 related myocardial injury [6]. Histopathologic, immunohistochemical, and ultrastructural analysis of transmural myocardial sections showed no definitive evidence of direct myocardial infection. COVID-19 cases frequently had cardiac fibrin microthrombi. The mechanism of fibrin micro thrombosis in the heart is unclear. The literature acknowledges a severe acute respiratory syndrome coronavirus 2 (SARS-CoV-2)-mediated systemic acquired coagulopathy, distinct from disseminated intravascular coagulation and thrombotic microangiopathy [7]. SARS-CoV-2 utilizes angiotensin-converting enzyme 2 (ACE2) as a receptor and given the importance of this single-pass transmembrane receptor in the cardiovascular system and the propensity of severe COVID-19 related illness among patients with cardiovascular comorbidity, it has been the subject of ongoing research [8].

The transmembrane enzyme ACE2 catalyzes the conversion of angiotensin II to angiotensin in vivo. Virally mediated downregulation of ACE2 receptors subsequently interferes with the receptor’s physiological responsibilities. Angiotensin, among its other properties, promotes antithrombotic homeostasis through binding to the Mas receptor on the platelet membrane. Mas receptor knockout animal models exhibit decreased bleeding time and increased thrombosis, which is corrected by the administration of angiotensin [8]. Based on these data, ACE2 receptor downregulation may promote a prothrombotic state through decreased circulating levels of angiotensin [9]. This effect is possibly potentiated by the hyper inflammation seen as a cellular immune response to viral invasion. High levels of interleukin-6, interleukin-2, interleukin-7, and tumor necrosis factor-alpha have been seen in patients with severe disease. The resultant homeostatic imbalance favors fibrin formation affecting several organ systems including the heart [10]. Myocarditis is present in one-third of patients with active and cleared COVID-19 but is usually limited in extent.

In a multi-hospital study of nearly 3,000 patients, it was noted that COVID-19-related systemic inflammation not only involved the lungs but had varying presentations of cardiac involvement. Interestingly, it was noted that myocardial injury in COVID-19 patients was associated with increased mortality risk up to three times. The same study noted myocardial injury in association with low-level elevation in troponin concentration and patients already having a history of cardiovascular disease were more likely to experience myocardial injury [11]. Based on pathophysiology of thromboembolic complications in patients with COVID-19 infection, the medical therapy should be targeted to prevent procoagulant activity using anticoagulation therapy [12]. 

## Conclusions

As knowledge about the pathophysiology of COVID-19 infection and its varied presentations grows, it is evident that multiple systems are involved in the disease process. While lungs appear to be the predominant organ involved, mortality exponentially rises with the involvement of myocardial injury. Further analysis of autopsy cases from COVID-19 patients with a special focus on evidence of myocardial injury is crucial to determining the frequency and impact of coronary microthrombi in the absence of coronary artery thrombosis in this patient population. Here, we present non-ST elevation MI in a COVID-19 infection patient who sustained regional wall motion abnormality as a complication from hypercoagulability and microthrombi from the potential disease processes. Clinicians may keep this possibility in mind while treating COVID-19 patients especially as the prognosis of patients without myocardial injury appears relatively favorable.
